# Bioprinted Multi-Cell Type Lung Model for the Study of Viral Inhibitors

**DOI:** 10.3390/v13081590

**Published:** 2021-08-11

**Authors:** Johanna Berg, Zia Weber, Mona Fechler-Bitteti, Andreas C. Hocke, Stefan Hippenstiel, Laura Elomaa, Marie Weinhart, Jens Kurreck

**Affiliations:** 1 Department of Applied Biochemistry, Technische Universität Berlin, Chair of Applied Biochemistry, 10623 Berlin, Germany; z.weber@tu-berlin.de (Z.W.); m.fechlerbitteti@tu-berlin.de (M.F.-B.); jens.kurreck@tu-berlin.de (J.K.); 2 Department of Internal Medicine/Infectious and Respiratory Diseases, Charité—Universitätsmedizin Berlin, Corporate Member of Freie Universität Berlin, Humboldt-Universität zu Berlin, and Berlin Institute of Health, 10115 Berlin, Germany; andreas.hocke@charite.de (A.C.H.); stefan.hippenstiel@charite.de (S.H.); 3 Department of Organic Chemistry, Institute of Chemistry and Biochemistry, Freie Universität Berlin, 14195 Berlin, Germany; laura.elomaa@fu-berlin.de (L.E.); marie.weinhart@fu-berlin.de (M.W.)

**Keywords:** bioprinting, human lung model, influenza A virus, LPS stimulation

## Abstract

Influenza A virus (IAV) continuously causes epidemics and claims numerous lives every year. The available treatment options are insufficient and the limited pertinence of animal models for human IAV infections is hampering the development of new therapeutics. Bioprinted tissue models support studying pathogenic mechanisms and pathogen-host interactions in a human micro tissue environment. Here, we describe a human lung model, which consisted of a bioprinted base of primary human lung fibroblasts together with monocytic THP-1 cells, on top of which alveolar epithelial A549 cells were printed. Cells were embedded in a hydrogel consisting of alginate, gelatin and collagen. These constructs were kept in long-term culture for 35 days and their viability, expression of specific cell markers and general rheological parameters were analyzed. When the models were challenged with a combination of the bacterial toxins LPS and ATP, a release of the proinflammatory cytokines IL-1β and IL-8 was observed, confirming that the model can generate an immune response. In virus inhibition assays with the bioprinted lung model, the replication of a seasonal IAV strain was restricted by treatment with an antiviral agent in a dose-dependent manner. The printed lung construct provides an alveolar model to investigate pulmonary pathogenic biology and to support development of new therapeutics not only for IAV, but also for other viruses.

## 1. Introduction

The human lung is the primary target organ for many viral and bacterial pathogens [1]. Lower respiratory tract infections caused by influenza A virus (IAV) infections are still a major cause of morbidity and mortality worldwide [2,3]. In addition to seasonal IAV strains, the emergence of pandemic IAV strains, such as H1N1 in 2009, results in even higher infectivity and pathogenicity [4,5], as they are often accompanied by an uncontrolled, severe response from the immune system [6,7,8]. Fulminant influenza infection of alveolar epithelial type II cells (AECII), the primary site of IAV replication [9,10], frequently leads to severe pneumonia [11]. In addition to IAV, bacteria can enter the deeper regions of the lung and infect alveolar epithelium [12,13]. Bacterial toxins such as lipo-poly-saccharide (LPS) or pneumolysin (PLY) induce the secretion of pro-inflammatory cytokines, including interleukin 1β (IL-1β), tumor necrosis factor α (TNFα) and IL-8, contributing to a severe immune reaction [14,15,16,17]. In addition to the alteration of the immune reactions of AECs, pathogenic infections influence cytokine release from alveolar macrophages, one of the major actors in pulmonary innate immunity. The resulting increase in migration and activation of macrophages facilitates this excessive inflammatory response [13,16,18].

Although animal models are frequently used to study the immune pathogenicity of IAV infections and have contributed significantly to our knowledge about IAV infection, they do not represent the physiology of the human lung and thus are limited in clinical translatability [19,20,21,22]. Some species, such as ferrets and guinea pigs, are susceptible to human IAV strains; however, their housing is comparatively elaborate and expensive, and they sometimes do not develop the expected symptoms [23]. Therefore, most research in the field is carried out in mouse models. However, mice are not naturally permissive to severe IAV infections, so viral adaption to the murine host is needed [23]. Another strategy to allow the use of animal models is the transgenic expression of human receptors to make them permissive for viral infection [24] as reported for the human angiotensin I converting enzyme 2 (ACE2) in case of SARS coronavirus-2 infection [25,26]. Furthermore, the translation of results obtained in mice to the human pathophysiology is hampered by significant differences between the murine and human immune systems [27]. Therefore, proper human models are needed to gain insight into the resulting immune reactions following IAV infection in humans [28].

The two- and three-dimensional culture of human cells and ex vivo models complement one another and together represent a viable alternative to mouse models for the study of pathogenic infections of the human lung [22]. While ex vivo cultivated lung tissue has been used successfully to identify target cells for IAV and identify immune signaling within the infection [10,29,30], its utility is restricted due to limited access and life span [31]. The 2D culture of cell lines and primary cells is an easy to handle, highly accessible tool, while on the other hand it has weaknesses in modeling in vivo conditions due to missing spatial interactions resulting from a physiological 3D microenvironment. Several research groups have proven that there are differences between the cellular responses of 2D and 3D cultured cells, demonstrating the relevance of the 3D environment for intercellular signaling and function [22,32,33,34,35]. Tissue engineering approaches hold great potential to generate in vitro models, which more closely mimic physiological conditions. Bioprinting enables precise spatial distribution of one or multiple cell types within a 3D matrix that allows for studying intercellular interactions in a manner that can be standardized and scaled [28,36,37,38].

In our first study aiming at the development of a bioprinted human lung model to investigate IAV infection, we printed the alveolar epithelial cell line A549 in a matrix composed of alginate, gelatin and Matrigel [39]. In our attempts to replace the use of components that are associated with the suffering of animals [40], we substituted Matrigel with human extracellular matrix (ECM) in the bioink for the generation of a liver model [41]. These organ models, however, were only composed of a single cell type, limiting the physiological relevance of the promising results.

The present study describes a substantially more sophisticated bioprinted lung model to better reflect human physiology and study the immune response, as well as the inhibition of viral infection. The model consisted of a top layer of epithelial A549 cells printed on a base composed of normal human primary lung fibroblasts (NHLFb) and macrophage-like THP-1 cells in an alginate/gelatin/collagen matrix. We cultured these models for up to 35 days, during which the viability of the cells remained stable. Starting between days 14 and 21 of the culture, the A549 cells in the models, printed together with fibroblasts and THP-1 cells, developed a clustered phenotype. This phenotype was not observed in models which only contained A549 cells. Furthermore, we stimulated the macrophage-like THP-1 cells in the model with LPS priming and a subsequent ATP pulse. This treatment resulted in the release of IL-1β. Finally, the model was used to study infection with the human seasonal IAV strain H3N2 and its inhibition by oseltamivir treatment.

## 2. Materials and Methods

### 2.1. Cell Culture and Virus Preparation

Human epithelial lung carcinoma cells (A549; ATCC, Manassas, VA, USA) and normal human primary lung fibroblasts (NHLFb; ATCC) were cultured using DMEM high glucose (Biowest, Nuaillé, France) supplemented with 10% fetal bovine serum (FBS; c.c.pro, Oberdorla, Germany), 1% 100× L-Glutamine (Biowest) and 1% penicillin/streptomycin (P/S; Biowest). Human monocytic leukemia cells (THP-1; DSMZ, Braunschweig, Germany) were cultured in DMEM low glucose (Biowest) supplemented with 10% FBS, 1% 100× L-Glutamine and 1% P/S. To induce maturation of monocytic THP-1 cells towards adherent macrophage-like cells, models were treated with 200 ng/mL phorbol 12-myristate-13-acetate (PMA, Sigma-Aldrich, Steinheim, Germany) for 24 h. Subsequently, printed samples were cultured in the medium without PMA for 48 h before infection and stimulation, respectively. Madine Darby canine kidney (MDCK) cells were propagated with MEM (Thermo Fisher Scientific, Waltham, MA, USA) supplemented with 10% FBS, 1% 100× GlutaMax (Thermo Fisher Scientific, Waltham, MA, USA) and 1% P/S. The seasonal human influenza A (H3N2) virus (A/Panama/2007/1999) was grown on the MDCK cells. Virus stocks were aliquoted, stored at −80 °C and titrated on the MDCK cells by plaque assays.

### 2.2. Bioink Preparation

Two bioinks were generated (Table 1). For bioink 1, gelatin type A (6.5% *w*/*v*) powder (Sigma-Aldrich, Steinheim, Germany) and sodium alginate (6.5% *w*/*v*) powder (Sigma-Aldrich, Steinheim, Germany) were dissolved in DMEM high glucose with supplements on a magnetic stirrer at 1250 min^−1^, at 37 °C overnight. The hybrid gelatin/alginate hydrogel was mixed with rat tail collagen I (Merck, Darmstadt, Germany), the respective cell types, CaSO_4_ (Carl Roth, Karlsruhe, Germany) and DMEM high glucose with supplements. The final cell-laden bioink 1 was composed of 3% *w*/*v* alginate, 3% *w*/*v* gelatin, 0.5 mg/mL rat tail collagen I, 0.045 M CaSO_4_, 2.5 × 10^7^ fibroblasts/mL and 3.5 × 10^6^ THP-1 cells/mL. Following CaSO_4_-driven initial cross-linking of alginate (8 min after mixing), bioink 1 was loaded into the printing cartridge. Bioink 2 was processed in the same manner as the first one, but its final composition was as follows: 2% *w*/*v* alginate, 3% *w*/*v* gelatin, 0.5 mg/mL rat tail collagen I, 0.03 M CaSO_4_ and 1.5 × 10^7^ A549 cells/mL.

### 2.3. Bioprinting

For the bioprinting process, the bioinks were extruded through a 22 G needle with either of the microextrusion printers INKREDIBLE+ or BioX (Cellink, Gothenburg, Sweden). The low viscosity bioink 1 was extruded at 10–15 kPa (11.5 mm/s); the high viscosity bioink 2 at 25–32 kPa (10 mm/s). The 3D construct was designed by the computer-aided design (CAD) software Rhinoceros5 (Robert McNeel & Associates, Barcelona, Spain). The printed constructs were submerged in 0.1 M CaCl_2_ (Carl Roth) to increase gelation of alginate and subsequently cultured in an incubator at 37 °C and 5% CO_2_ in DMEM high glucose with supplements, and 5 mM sodium citrate, as well as 20 mM CaCl_2_ for up to 35 days.

### 2.4. Rheology

The viscoelastic behavior of the 3D printed constructs was analyzed using a Kinexus lab+ oscillating rheometer (Malvern Panalytical Ltd., Malvern, UK) with an active hood Peltier plate cartridge (Malvern Panalytical Ltd., Malvern, UK). Printed samples were cultured as described above and tested at the indicated time points. The elastic modulus of the wet 3D printed hydrogels was recorded within a viscoelastic regime by running a frequency sweep of 0.1–10 Hz at 0.1% shear strain. The measurements were carried out at 37 °C using an 8 mm parallel plate geometry.

### 2.5. Cell Viability

The metabolic activity of the bioprinted cells was determined using the tetrazolium hydroxide salt (XTT) assay according to the manufacturer’s instructions (AppliChem, Darmstadt, Germany) at the indicated time points. Briefly, printed cell-laden constructs were cultured for 7, 28 or 35 days (37 °C, 5% CO_2_). XTT reagent (1 mg/mL) and phenazine methosulphate (PMS; 100 µM) were diluted in RPMI w/o phenol red (Biowest), added to the samples and incubated for 4 h (37 °C, 5% CO_2_). The absorbance of the resulting solution was measured spectrophotometrically at A450 nm (TriStar Multimode Reader LB942, Berthold Technologies, Bad Wildbad, Germany) with a reference of A620 nm. Cell-laden constructs incubated in culture medium supplemented with 10% Triton-X-100 (Carl Roth) were used as the lysis control. Values were normalized to lysis controls.

For the cell viability assay (Viability/Cytotoxicity kit, Thermo Fisher Scientific, Waltham, MA, USA), 3D printed samples were stained with 2 µM calcein-AM and 4 mM ethidium homodimer-1 diluted in RPMI w/o phenol red for 30 min (37 °C, 5% CO_2_). The samples were analyzed by fluorescence microscopy (Zeiss Observer Z1 microscope; Zeiss, Jena, Germany).

### 2.6. LPS Stimulation

Following the induction of the maturation of monocytic THP-1 cells towards the adherent macrophage-like type using PMA, samples were cultured for another 48 h without PMA. Afterwards, samples were challenged with 1 µg/mL lipopolysaccharide (LPS) from Escherichia coli O111:B4 (Sigma-Aldrich, Steinheim, Germany) for 16 h followed by 30 min of stimulation with 10 µM adenosine triphosphate (ATP, Alfa Aesar, Ward Hill, MA, USA). Supernatants were collected to quantify the release of IL-1β and IL-8.

### 2.7. Viral Growth Assay in the Presence of Oseltamivir

All viral assays were carried out with the seasonal IAV strain Pan/99(H3N2). For virus inhibition experiments, the culture medium was replaced with media containing either 0, 0.1, 0.5, 1 or 10 µM oseltamivir carboxylate (MedChemExpress, Monmouth Junction, NJ, USA) two hours before infection. Subsequently, bioprinted samples were washed three times with 1xHBSS and inoculated with 4 × 10^5^ PFU for 1.5 h at room temperature. The solution was then removed and replaced by DMEM high glucose supplemented with 2% FBS, 2 mM Glutamine, 1% penicillin-streptomycin, 0.2 mg/mL TPCK-trypsin (Thermo Fisher Scientific, Waltham, MA, USA) and indicated concentrations of oseltamivir. Samples were incubated for 48 h (37 °C; 5% CO_2_). Supernatants were taken 1, 16, 24 and 48 h after infection and titrated on MDCK cells by a standard plaque assay to determine the infectious viral yield.

### 2.8. Immunostaining

Bioprinted specimens were fixed in 3.7% formalin (Carl Roth) overnight at 4 °C and treated with a commercial tissue clearing kit (abcam, Cambridge, UK) according to the manufacturer’s instructions. Samples were analyzed for expression of cyclophilin B (abcam), pan-cytokeratin (Pan-CK; Santa Cruz Biotechnology, Inc., TX, USA), fibroblast specific protein 1 (FSP-1; LifeSpan BioSciences, Seattle, WA, USA) and pro-surfactant protein C (pro-SPC; Millipore, Billerica, MA, USA) Afterwards, the sample sections were incubated with corresponding Alexa 546- and Alexa 488-conjugated secondary antibodies (Invitrogen, Carlsbad, CA, USA), and nuclear counterstaining was performed with 4′,6-diamidino-2-phenylindole (DAPI, Thermo Fisher Scientific, Waltham, MA, USA). For imaging the samples were finally mounted in clearing buffer II (abcam) in a 3.5 mm silicon imaging chamber (abcam) and analyzed with the Observer Z1 microscope (Zeiss Observer Z1 microscope; Zeiss).

### 2.9. ELISA

The interleukins 8 (IL-8), 1β (IL-1β) and 29 (IL-29/IFN λ1) released from the cells were quantified in the culture supernatant using the Human IL-8 ELISA set (BD Biosciences; Franklin Lakes, NJ, USA), Human IL-1β and Human IL-29 ELISA Ready-SET-Go! Enzym-Linked Immunoabsorbant Assay (ELISA) kits (Thermo Fisher Scientific, Waltham, MA, USA), respectively. This was performed according to the manufacturer’s instructions, at an absorbance of A450 nm with a reference of A620 nm (Sunrise absorbance microplate reader, Tecan, Männedorf, Switzerland).

### 2.10. Statistical Analysis

The statistical evaluation of the experiments was performed using a one-way analysis of variance (ANOVA) or two-way ANOVA with Tukey´s correction (GraphPad Prism 6, GraphPad Software, Inc.; La Jolla, CA, USA). Each set of cell-laden experiments was repeated at least three times. Data are represented as mean ± standard error of the mean (SEM), *p* values are considered significant by * *p* ≤ 0.05; ** *p* ≤ 0.01; *** *p* ≤ 0.001; **** *p* ≤ 0.0001.

## 3. Results

### Generation and Characterization of the Bioprinted Three Cell Type Lung Model

The first task on the way towards generating a multi-cell type lung model was to optimize the bioink composition. To this end, a basic composition of 3% alginate and 3% gelatin was blended with 0.25, 0.5 or 1 mg/mL collagen, respectively, and mixed with primary human lung fibroblasts. The bioink was used to print a waffle-shaped tissue model, which is widely utilized in the bioprinting field, e.g., [42,43,44] by pneumatic extrusion printing. The 3D construct was then cultured for 14 days and metabolic activity was measured as an indicator for cell viability (Figure S1A). No significant differences were detected for the collagen concentrations tested until day four of the culture. From day seven onwards, cells cultured in the absence of collagen had a significantly lower metabolic activity than those in constructs containing 0.5 mg/mL and 1 mg/mL collagen.

Next, the rheological properties of models printed without or with either 0.5 mg/mL or 1 mg/mL collagen were analyzed. Even though the elastic (Figure S1B) and viscous (Figure S1C) moduli were quite similar for different collagen concentrations, the elastic modulus of the bioink containing 1 mg/mL collagen was not as stable as that of the bioink containing 0.5 mg/mL collagen over the culture period of 14 days. In addition, printing of the bioink containing 1 mg/mL collagen was hampered by frequent nozzle clogging and insufficient shape fidelity in the process of cross-linking the alginate. Thus, although the viability of cells cultured in constructs with 1 mg/mL collagen was slightly higher than that of the ones cultured with lower collagen concentrations, all further experiments were performed with a bioink consisting of 3% alginate, 3% gelatin and 0.5 mg/mL collagen for optimal shape fidelity and reproducibility.

During the printing process, the components of the bioink, such as alginate, protect the cells from shear stress by encapsulation. For subsequent cultivation, however, interactions between the cells are desirable to mimic their natural physiology. To this end, alginate cross-linking can be reversed by the addition of sodium citrate [45]. Increasing concentrations of sodium citrate (0, 2.5, 5, 10 or 20 mM) were added to the medium and fibroblast-laden constructs were cultured for 28 days. The highest levels of metabolic activity were observed for constructs cultured in the presence of 5 mM and 10 mM sodium citrate (Figure S1D). The addition of 20 mM sodium citrate resulted in dissociation of the printed constructs starting between day 7 and 14 post printing. To confirm the reproducibility of these results, the experiment to compare metabolic activity of the cells in constructs cultured in the absence or presence of 5 mM sodium citrated was carried out eight times. As can be seen in Figure S1E, stable and significant differences were observed for the two cultivation conditions over a time range of up to 28 days.

**Figure 1:**
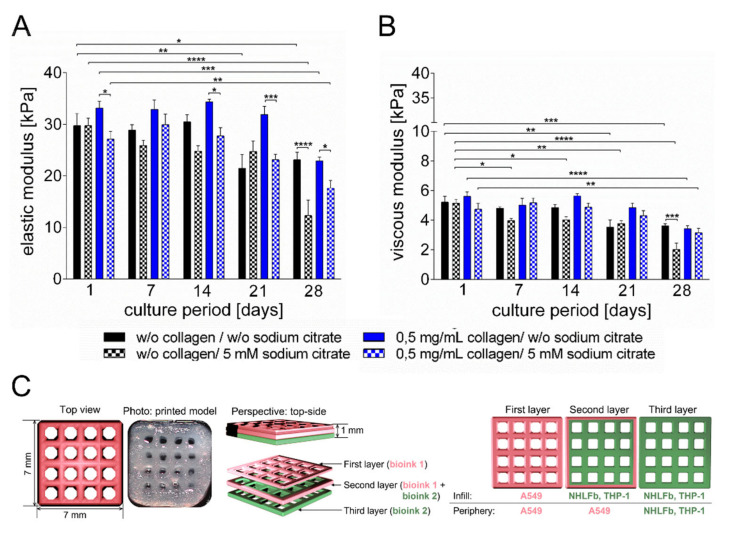
Rheologic characterization of the printed tissue models cultured in the presence or absence of sodium citrate. (**A**) The elastic modulus was measured at a frequency of 1 Hz and 0.1% shear strain at 37 °C at the indicated time points. (**B**) Shear modulus of printed constructs at increasing frequencies (0.1–10 Hz). (**C**) Schematic representation of the multi-layer structure and photograph of the printed model. Results are shown as mean ± SEM of three independent experiments. * *p* ≤ 0.05, ** *p* ≤ 0.01, ***, *p* ≤ 0.001, **** *p* ≤ 0.0001.

To follow changes in bioink properties, further rheological characterizations were carried out, and the storage and loss moduli of the printed constructs were measured weekly at a shear frequency of 1 Hz and a shear stress of 0.1 % (Figure 1A,B). For samples containing 0.5 mg/mL collagen, no significant differences of the viscous modulus (loss modulus) were observed for cultivation in the presence or absence of sodium citrate over the cultivation period of 28 days (Figure 1B). In contrast, the elastic modulus had lower values for the constructs cultured in the presence of 5 mM sodium citrate compared to those maintained in the absence of sodium citrate (Figure 1A). While this effect was rather modest at first, it became more pronounced and statistically significant after day 14 of cultivation. Importantly, the shape fidelity of the printed constructs containing 0.5 mg/mL collagen also remained intact in the presence of sodium citrate.

After optimizing the bioink and culture conditions (0.5 mg/mL collagen, 5 mM sodium citrate), the final model containing three different cell types was generated. The base contained fibroblasts and THP-1 cells and made up around 65% of the volume of the complete model. The infill of the top layer and 67% of the periphery of the complete construct contained A549 cells, which made up the remaining 35% of the total construct volume (Figure 1C). The ratio of monocytes (THP-1) and epithelial cells was set to 1:4. THP-1 cells were printed as monocytic cells, and maturation towards macrophage-like characteristics was induced by adding 200 ng/mL PMA to the culture media 72 h before LPS/ATP stimulation or infection with the influenza virus. After 24 h of PMA treatment, PMA-containing media was exchanged for PMA-free media. Afterwards, the cells could rest for 48 h till further treatment.

**Figure 2:**
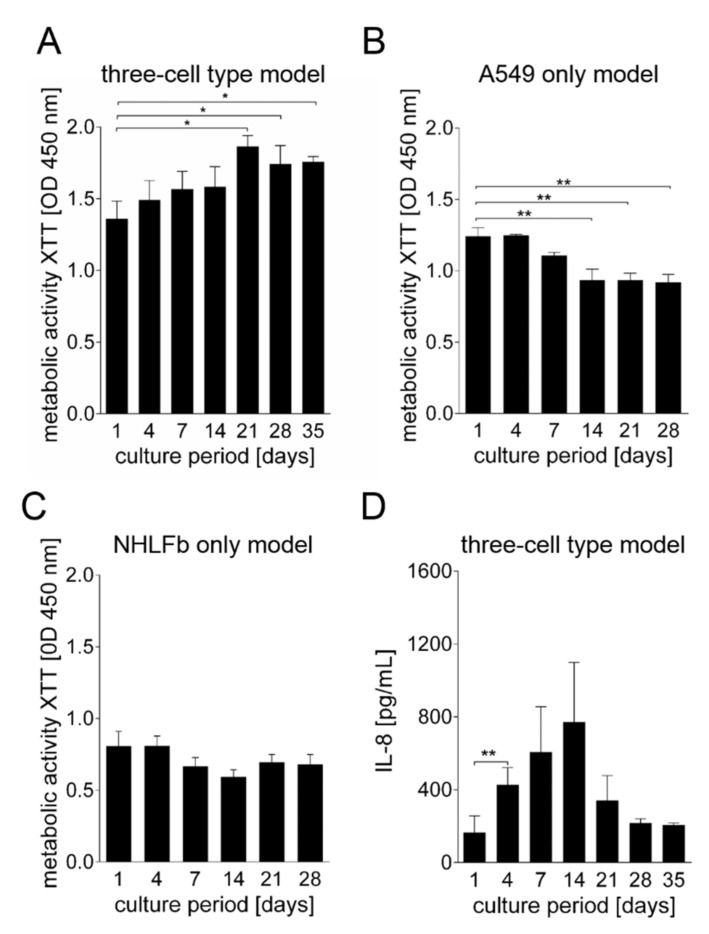
Metabolic activity and secretion of IL-8 from the bioprinted models. (**A**–**C**) Metabolic activity as determined by the tetrazolium hydroxide salt (XTT) assay at the indicated time points post-printing of models containing (**A**) A549 cells, THP-1 cells and human lung fibroblasts (NHLFb), as well as models containing either (**B**) only A549 cells or (**C**) only NHLFb. All models were printed in alginate/gelatin/collagen bioinks. (**D**) Quantitative enzyme-linked immunosorbent assay (ELISA) analysis of IL-8 from the three cell type model measured at indicated time points post-printing. Results are shown as mean ± SEM of five (**A**,**D**) or three (**B**,**C**) independent experiments. * *p* ≤ 0.05; ** *p* ≤ 0.01.

The constructs containing A549 cells, fibroblasts and THP-1 cells were cultured for 35 days. Viability was confirmed by weekly measurements of XTT metabolization and cell viability staining. Metabolic activity remained high throughout the entire culture period lasting 35 days. Starting at day 21, there was a slight but significant increase of the metabolic activity (Figure 2A).

For comparison, the metabolic activities of models printed either only with A549 cells (1.5 × 10^7^ cells/mL) or fibroblasts (2.5 × 10^7^ cells/mL) were also measured. Direct comparison of the XTT values was hampered by the different cell numbers used in each case, but it was interesting to see that A549 cells (Figure 2B) had higher XTT values than fibroblasts (Figure 2C), although they were printed at lower cell numbers per mL. Even more important is the trend of the metabolic activity over time: while, as described above, the XTT values of the model containing three cell types increased over time, reaching statistical significance by day 21, they remained constant in the case of the pure fibroblasts and declined in the case of the A549 cells alone. This finding indicates that interactions between different cell types are necessary for growth and survival in long-term cultivation.

The IL-8 release was determined to investigate cellular stress during the long-term culture of the multi-cell type model (Figure 2D). These analyses revealed increasing levels of IL-8 secretion until day 14 of the culture, followed by a decrease of the IL-8 values. This course of IL-8 secretion indicates that cellular stress was more pronounced in the early phase of cultivation, while the cells reached a more stable status at later time points. For this reason, infection experiments with IAV were carried out starting on day 21 of the culture.

**Figure 3:**
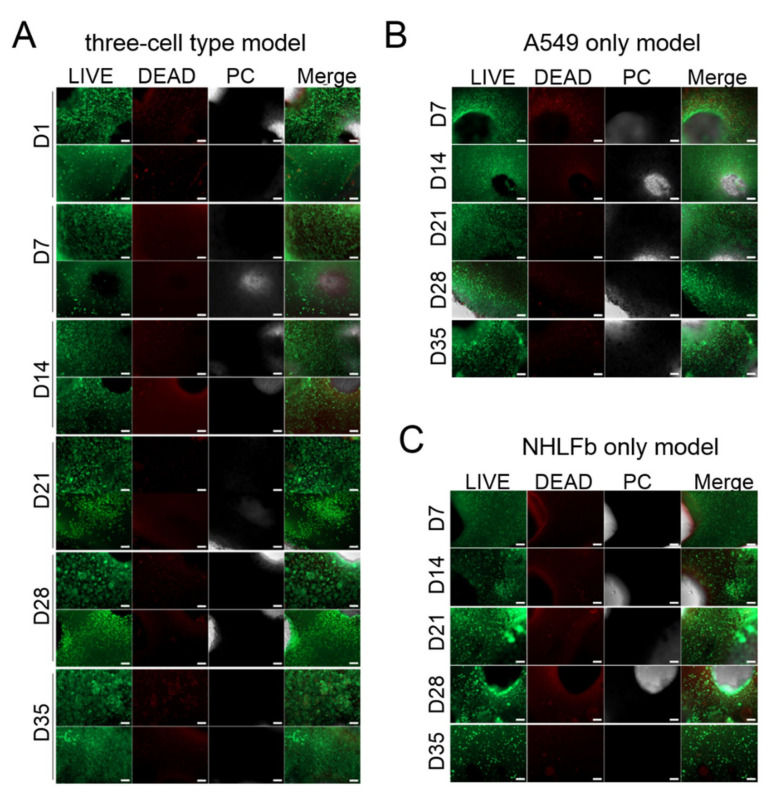
Cell viability staining of bioprinted lung models. Qualitative viability staining of living and dead cells in models containing (**A**) A549 cells, THP-1 cells and human lung fibroblasts (NHLFb), as well as in models containing either (**B**) only A549 cells or (**C**) only NHLFb printed in an alginate/gelatin/collagen bioink after indicated time points of cultivation post printing using calcein-AM (live in green) and ethidium homodimer-1 (dead in red). Scale bar: 200 µm.

Cell viability assays did not reveal a substantial increase in dead cells over the culture period, neither for individually printed A549 cells or fibroblasts nor for the three cell type model (Figure 3). Starting between days 14 and 21 of the culture, the morphology of the cells in the three cell type model started to change (Figure 3A). The A549 cells formed clusters of cells in an egg-like shape. Fibroblasts initially had a round shape resulting from the encapsulation in bioink components. At later time points, they also started to change their morphology towards the spindle-like structure characteristic for fibroblasts in vivo. However, not all fibroblasts left encapsulation, and compared to the morphology changes of A549 cells, fibroblast morphology changes took longer and were not as prominent as those observed for the A549 cells.

To see whether the observed phenotype of the A549 cells is influenced by co-printed fibroblasts/THP-1 cells, samples containing only A549 cells were printed and analyzed by cell viability staining (Figure 3B). Although calcein metabolism revealed a high percentage of living cells during the 35 days of culture, clustering, as seen in the co-printing with fibroblasts, could not be detected. Likewise, the morphological change of fibroblasts towards the spindle-like shape was less pronounced in the single cell type model compared to the three cell type model (Figure 3C).

**Figure 4:**
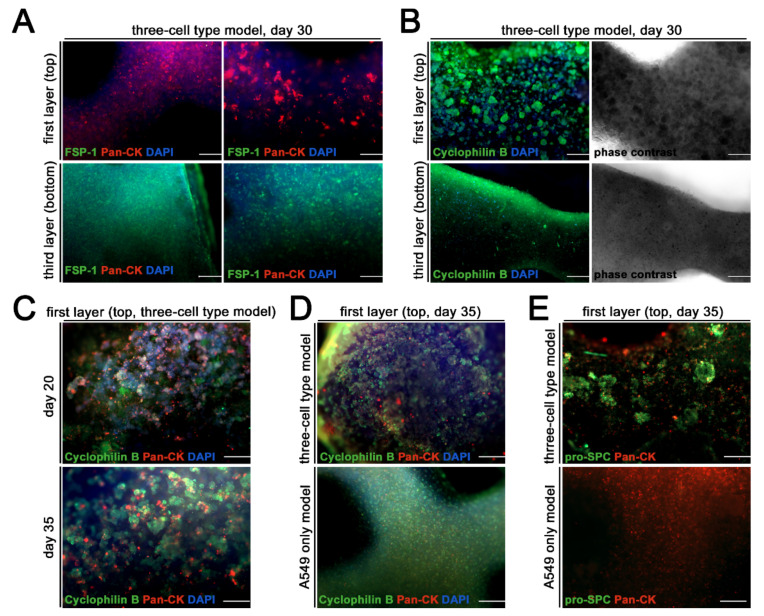
Differential protein expression and morphology between printed cells. Bioprinted models containing either A549 cells, THP-1 cells and human lung fibroblasts or models containing only A549 cells were cultured for indicated time points, fixed, immunohistochemically labelled, cleared and analyzed by fluorescence microscopy. Type of model, culture time, side of view as well as stained proteins are mentioned in the respective panels (**A**–**E**). Nuclear counter staining was performed using DAPI (blue channel). Scale bar involves (**A**) (left column), (**B**,**D**,**E**): 200 µm; (**A**) (right column) and (**C**): 100 µm.

To analyze the expression of cell markers, as well as the morphology and organization of the bioprinted cells in the model, they were immunostained with specific antibodies and imaged by fluorescence microscopy. In accordance with the printing strategy, a specific distribution of the cell types could be seen. Models containing A549 cells, THP-1 cells and fibroblasts showed expressions of Pan-CK (red channel) in the first layer (top), which contained only A549 cells, but no signals from the fibroblast-specific protein FSP-1 (green channel). In contrast, the third layer (bottom) showed an expression of FSP-1, but not of Pan-CK (Figure 4A), which is characteristic of fibroblasts. For visualization of the cell morphology, the three cell type model was also stained using an antibody against cyclophilin B (green channel) (Figure 4B). In line with observations resulting from the cell viability staining, the A549 cell-containing top layer showed “egg-like” clustering of the epithelial cells, whereas the fibroblast (and THP-1 cells) in the bottom layer remained quite planar. The phase contrast image also showed the clustering of the A549 cells in the top layer (Figure 4B). The formation of the “egg-like” organization of the A549 cells in the top layer started around day 20 of cultivation. They expressed Pan-CK (red channel) and cyclophilin B (green channel) at both time points, day 20 and day 35 of the culture. The expression pattern of cyclophilin B, revealed that clustering of the A549 cells continued to intensify up to the end of the culture on day 35 (Figure 4C). The comparison of the three cell type model with the model containing only A549 cells revealed that A549 cells printed without fibroblasts/THP-1 cells showed no “egg-like” clustering after 35 days, mainly indicated by the expression pattern of cyclophilin B (green channel), but also of Pan-CK (red channel) (Figure 4D). To see whether the co-cultivation of A549 cells, fibroblasts and THP-1 cells induced the expression of the typical AECII cell marker pro-SPC, both the three cell type model, and the A549-only model were stained using an antibody against pro-SPC (green channel) and Pan-CK (red channel) after 35 days of culture. In this case, only the A549 cells in the three cell type model showed an expression of pro-SPC, and even then it was restricted to areas of “egg-shape” organized cell clusters (Figure 4E). Individually printed A549 cells, which did not form clusters (Figure 4D,E), also showed no expression of pro-SPC (Figure 4E).

Following the basic physiological characterization of the bioprinted lung model, its response to infectious stimuli was investigated. Innate immunity is the first line of host defense. The activation of inflammasomes, intracellular multimeric protein complexes, is an essential part of inflammatory pathways, which results in activation of the inflammatory caspase-1. Active capsase-1, in turn, cleaves downstream targets such as pro-interleukin-1β (pro-IL-1β) into its mature and biologically active form IL-1β. For the NLRP3 inflammasome, a two-signal model was proposed, composed of a priming signal which activates NF-κB and subsequently upregulates NLRP3 and pro-IL-1β, and an activation signal which finally activates NLRP3 [46,47].

**Figure 5:**
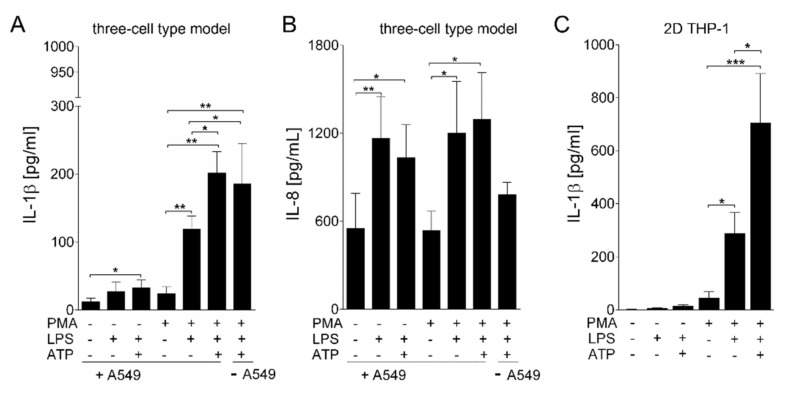
LPS-induced release of IL-1β from bioprinted multi-cell type lung models. (**A**,**B**) A549 cells were printed on top of a base containing THP-1 cells and NHLFb (fibroblasts). Additionally, constructs containing only NHLFb and THP-1 cells were printed (- A549). The printed constructs were cultured for 21 days. For maturation of THP-1 cells towards macrophage-like cells, the constructs were treated with 200 ng/mL PMA for 24 h. After a further 48 h, constructs were stimulated with 1 µg/mL lipopolysaccharide (LPS) for 16 h, followed by 30 min of treatment with 10 µM adenosine triphosphate (ATP). Centrifuged supernatants were assayed for the release of (**A**) IL-1β and (**B**) IL-8 by enzyme-linked immune assays. (**C**) THP-1 cells were seeded in 24-well plates and matured with PMA (100 ng/mL, 24 h) towards macrophage-like cells. After 48 h of culture without PMA, THP-1 cells were stimulated with LPS (1 µg/mL, 16 h), followed by 10 min of treatment with 5 µM ATP. Centrifuged supernatants were assayed for the release of IL-1β by enzyme-linked immune assays. Results are shown as mean ± SEM of three (**A**,**B**) and five (**C**) independent experiments.* *p* ≤ 0.05; ** *p* ≤ 0.01; *** *p* ≤ 0.001.

To test whether the matured macrophage-like THP-1 cells in the printed model react towards bacterial stimuli in terms of the release of proinflammatory IL-1β, the model was primed with 1 µg/mL of the Gram-negative bacterial toxin LPS for 16 h as a first stimulus. For activation of the NLRP3 inflammasome, the model was subsequently challenged with ATP (10 µM) for 30 min. Subsequently, IL-1β release was quantified in the supernatants by ELISA (Figure 5A). Interestingly, even in models, in which the maturation of THP-1 cells was not induced, a small amount of IL-1β was released following LPS/ATP stimulation. The level of IL-1β released in these experiments was significantly higher than that released from models that had not been challenged with the infectious stimulus. Nevertheless, IL-1β release from the models, in which THP-1 maturation was induced by PMA treatment, was increased substantially. Although a two-signal mode for NLRP3 activation has been proposed [46,47], a strongly increased release of IL-1β was detected already after just the priming signal by LPS, though only an average of 119 pg/mL. The second stimulus, ATP, further increased IL-1β release by approximately another 42% (average 202 pg/mL). Compared to the naïve models (not treated with PMA) stimulated with LPS/ATP (average 33 pg/mL), activation of THP-1 cells into their macrophage-like state led to an 83% increase in the release of IL-1β. Samples printed without A549 cells were also challenged with PMA, LPS and ATP. In these models, IL-1β release was only marginally lower (approximately 8%) than in the three cell type model, confirming that the THP-1 cells are the major source of IL-1β. Furthermore, IL-8 expression was not dependent on PMA treatment.

Constructs challenged with LPS alone and with LPS and ATP, respectively, released similar amounts of IL-8 ranging between 1050 to 1250 pg/mL, independent of PMA pre-treatment (Figure 4B). Furthermore, no substantial differences between one signal (LPS) and two-signal (LPS+ATP) stimulation could be detected. Compared to IL-1β, which was not released in the absence of stimulation, the baseline of IL-8 release from untreated samples was comparatively high, at approximately 550 pg/mL. Models printed without A549 cells released IL-8 (average 780 pg/mL) following stimulation with LPS and ATP; however, the expression was reduced compared to models containing A549 cells (Figure 5B). Thus, A549 cells contribute to the levels of IL-8 released by the model, but not to the level of IL-1β released.

For comparison, fibroblasts, THP-1 cells and A549 cells were seeded onto 2D monoculture plates and challenged with LPS and ATP. Matured THP-1 cells cultured in 2D conditions released higher absolute amounts of IL-1β but showed the same induction pattern found in 3D cultures (Figure 5C). In the 2D THP-1 culture, PMA treatment resulted in a substantial and statistically significant increase of IL-1β release following LPS stimulation, which was again increased by the second stimulus with ATP. For both other cell types, fibroblasts and A549 cells, no release of IL-1β could be measured (data not shown).

**Figure 6:**
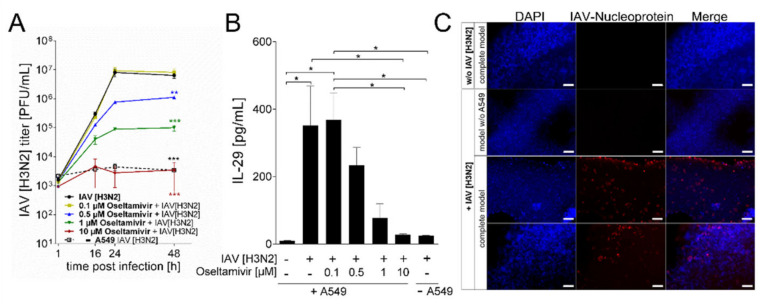
Oseltamivir inhibition of IAV replication in bioprinted lung models. The A549 cells were printed on top of a base containing THP-1 cells and NHLFb (fibroblasts). Additionally, constructs containing only NHLFb and THP-1 cells were printed (- A549). The printed constructs were cultured for 21 days. For maturation of THP-1 cells towards macrophage-like cells, constructs were treated with 200 ng/mL PMA for 24 h. (**A**,**B**) After a further 48 h, constructs were infected with 4 × 10^5^ PFU/mL of human IAV [H3N2] and treated with different concentrations of oseltamivir. (**A**) Supernatants were taken 1, 16, 24 and 48 h post infection and titrated on MDCKII cells to determine IAV titers. (**B**) Centrifuged supernatants 48 h post IAV infection were assayed for release of IL-29 by enzyme-linked immune assays. Results are shown as mean ± SEM of three independent experiments. * *p* ≤ 0.05; ** *p* ≤ 0.01; *** *p* ≤ 0.001. (**C**) Immunohistochemical analysis of IAV infection. Constructs were infected with 1 × 10^6^ PFU/mL of IAV for 24 h. Afterwards, samples were fixed and stained for viral nucleoprotein (red) and nuclei (DAPI, blue). Staining was analyzed by fluorescence microscopy. Scale bar: 200 µm.

The final aim of this study was to use the newly developed lung model for virus inhibition assays. To this end, printed models were infected with 4 × 10^5^ PFU/mL of the IAV strain A/Panama/1999 (H3N2) and treated with increasing concentrations of the neuraminidase inhibitor oseltamivir (0, 0.1, 0.5, 1 or 10 µM). To determine inhibition of viral growth by the antiviral drug, supernatants were taken 1 h, 16 h, 24 h and 48 h post infection and titrated on MDCK cells. The antiviral treatment resulted in a clear dose-dependent inhibition of viral growth (Figure 6A). The lowest oseltamivir concentration tested, 0.1 µM, was insufficient to reduce viral growth, as titers were comparable to those of IAV infected samples that had not been treated with the antiviral drug. A clear reduction of viral replication was seen with 0.5 µM oseltamivir. The viral titers were reduced from nearly 8 × 10^6^ PFU/mL to 7.67 × 10^5^ PFU/mL at 24 h post infection and from 6.4 × 10^6^ PFU/mL to 1.12 × 10^6^ PFU/mL at 48 h post infection. The inhibitory effect became even more pronounced at higher oseltamivir concentrations. At a concentration of 1 µM oseltamivir, the virus titers were reduced to 8.9 × 10^4^ PFU/mL at 24 h and 1 × 10^5^ PFU/mL after 48 h of infection, respectively. At the highest dose of 10 µM oseltamivir, virus replication was almost entirely suppressed. The initial virus load of 9.46 × 10^2^ PFU/mL, determined 1 h post infection, increased only slightly to 2.8 × 10^3^ PFU/mL after 24 h and 3.46 × 10^3^ PFU/mL after 48 h, respectively. In constructs consisting only of THP-1 cells and fibroblasts, no increase of the virus titer was observed, confirming that IAV can only replicate in alveolar epithelial cells.

To further characterize the immune response of the printed lung modes, we measured the release of IL-29 in the supernatants 48 h post infection with IAV (Figure 6B). In line with the virus replication assays, untreated models and models treated with a very low oseltamivir concentration of 0.1 µM secreted high levels of IL-29. At a concentration of 0.5 µM oseltamivir, a reduction in IL-29 release was observed, which, did not reach statistical significance. This finding is in line with the intermediate reduction of viral growth as determined by plaque reduction assays at this concentration. Treatment of the infected models with 1 µM and 10 µM oseltamivir caused a significant reduction of IL 29 expression. As a control, models printed without A549 cells were also infected with IAV. No significant amount of IL-29 was secreted, indicating again that epithelial cells are required for infection and replication of IAV.

The essential function of alveolar epithelial cells for IAV infection experiments was further confirmed by an immunohistochemical analysis (Figure 6C). Printed models with or without epithelial A549 cells were infected with IAV at 10^6^ PFU/mL for 24 h. Subsequently, samples were fixed and stained against the influenza nucleoprotein (NP). The images clearly demonstrate that only constructs printed with A549 cells promoted IAV infection, as indicated by the red fluorescence originating from the viral nucleoprotein.

## 4. Discussion

Three-dimensional printing technologies are already in common use in basic and clinical research projects to address topics like tissue repair (regenerative medicine), cancer pathogenesis and the overall goal of using bioprinting for fabrication of transplantable organs or organ parts [48,49]. Within the last few years, bioprinting has also become interesting for pharmaceutical applications in terms of drug testing and high-throughput screening. With the recent viral outbreaks of SARS-CoV-2, SARS, MERS, Zika and the influenza outbreaks of 2009 (H1N1) and 2013 (H7N9), human 3D lung models in general [50,51], and bioprinting technologies in particular, have gained significantly more traction in research devoted to combating infectious diseases [52]. Here, we present a bioprinted lung model that is composed of epithelial A549 cells printed on a base of primary lung fibroblasts and monocytic THP-1 cells. The model responded in the expected manner to a challenge with the bacterial toxin LPS and ATP, as well as towards infection with a seasonal IAV strain. In addition, IAV infection was restricted in a dose-dependent manner with the antiviral agent oseltamivir.

After seeding in cell culture plates, maintenance of healthy 2D cultures of A549 cells or fibroblasts only last a few days before cells start to overgrow each other, resulting in stressful conditions for the lower cell layers or complete death of the cells. In the printed model, the cells could be cultured for a prolonged time. They were kept for 35 days without a significant decrease in viability in the three cell type model. In contrast, models containing only A549 cells started with a similar viability, which decreased beginning on day seven after printing. After 14 days of the culture, the decrease became significant. The printed fibroblasts, on the other hand, remained viable during the whole culture period. Although they possessed a generally lower rate of metabolic activity, compared to the bioprinted A549 cells, they only showed a small drop of viability within the first 14 days of the culture, which nearly vanished starting at day 21 of the culture. In the three cell type model, a slight increase in viability was even observed, which became significant at 21 days post printing. Concomitantly, after 21 days of culture, the levels of secreted IL-8 decreased in the model. While IL-8 expression is primarily associated with oxidative stress, it can also be associated with high mechanical stress, which the cells are subjected to during the printing process. However, the release of IL-8 increased steadily until day 21, before it began decreasing again down to levels comparable to those on day one. It is thus unlikely that IL-8 release is triggered solely by the mechanical stress of the printing process and is most likely caused by encapsulation by alginate, a component of the bioink. This is supported by the time course of cell growth in the presence of sodium citrate in the culture media which dissolves alginate cross-linking. Under these conditions, the viability of the printed cells started to improve between weeks two and three of the culture compared to cells cultured in the absence of sodium citrate.

Furthermore, morphological changes of the A549 cells in the three cell type model became visible, starting 21 days post printing. These changes were not observed in models containing only A549 cells. It can be assumed that the fibroblasts, which are the main producers of ECM components in the lung as well as key regulators of ECM protein homeostasis [53], facilitated the organization and polarization of the A549 cells. This polarization of carcinoma-derived epithelial cells from different regions of the lung was observed following the culture on permeable membranes; however, the extent of differentiation and polarization depended on various aspects of culture conditions, including time and cell type [54]. Cooper et al. compared freshly isolated alveolar epithelial type II cells with A549 cells cultured for 25 days by a microarray analysis and found increasing numbers of up- and down-regulated genes shared between both cell types, thereby identifying conditions promoting the AECII differentiation characteristics in A549 cells [55]. In the bioprinted model, the morphological changes of A549 cells were first observed 21 days after the beginning of the culture (similar to the report by Cooper et al.) and the process went on for the following weeks. In addition to stabilizing the long-term culture of the three cell combination, fibroblasts and THP cells might also modulate the morphological changes. Immunohistochemical staining of the models for pro-SP-C demonstrated expression, albeit weak, of this AECII marker in A549 cells in the three cell type model, whereas no expression of pro-SP-C was detected in models containing only A549 cells (Figure 4). The pro-SP-C expression was restricted to areas of A549 cells which underwent reorganization into egg-like clusters, whereas flat, non-clustered areas of A549 cells were only positive for cytokeratin, but not pro-SP-C. This finding indicates that the organization state of the cells is another factor influencing the physiological characteristics of A549 cells, in addition to the duration of culture and co-cultivation with other cell types.

For in vitro models in alveolar research, AECII cells are currently considered the physiologically most relevant cells; however, their use is restricted by the limited availability of human lung tissue. Moreover, they differentiate towards the AECI phenotype within several days, limiting their use to short-term experiments [56]. Therefore, it is desirable to determine parameters which promote AECII differentiation characteristics in the printed A549 cells. To this end, further research is needed to identify these factors and trigger them in a specific manner to generate reproducible, reliable alveolar models. The matrix base, in this case composed of fibroblasts and THP-1 cells, bioprinted in the collagen-containing bioink could serve as a membrane capable of polarizing epithelial lung cells. Numerous factors such as cell types, cell number, culture time, culture conditions, media composition, printing process, bioink composition and shape fidelity will contribute to the structural and physiological features and interact in a complex manner. Although some parameters directly influence each other, for example, the breakdown of encapsulation by supplementation with sodium citrate is known to increase cell viability, the impact of other parameters cannot be easily predicted, and direct assessment of individual factors is complicated by complex interactions.

In our study, we differentiated the printed monocytes towards macrophage-like THP-1 cells by treatment with PMA. Subsequently, the constructs were primed with LPS to activate the transcription of pro-IL-1β and then treated with ATP to provide a second pro-inflammatory signal. The ATP stimulus induces the cleavage of pro-IL-1β into active IL-1β through NLR-containing inflammasomes and the activation of caspase-1 [46,47]. Although co-stimulation of the models with LPS and ATP resulted in higher release of IL-1β, treatment with LPS alone was sufficient to trigger the release of active IL-1β. To exclude the possibility that this might be an artefact resulting from the printing procedure itself, 2D cultured THP-1 cells were treated in a similar manner with LPS and ATP. In these experiments, treatment with just LPS also resulted in the release of IL-1β, and unlike in the 3D constructs, IL-1β substantially increased after the second stimulus with ATP.

In our previous study, we used a simple bioprinted model consisting only of A549 cells, which we infected with IAV a few days post printing [39]. Here, we present a much more sophisticated model consisting of a layer of fibroblasts and THP-1 cells that were overlaid with alveolar epithelial A549 cells. This model is thus a step towards an improved representation of the physiological architecture. An important advantage of the multi-cell model, compared to simpler models or standard 2D cultures, is the high cell viability in long-term cultivation experiments lasting up to 35 days. From the third week of the culture, the morphology of A549 cells changed towards an “egg-shaped” clustering. The long-term cultivation gives the experimenter a wider window of research opportunities and may be advantageous for co-infection experiments and research addressing tissue repair or fibrosis, as well as for long-term studies of toxic compounds or drugs.

Immunohistochemical staining clearly showed that the epithelial A549 cells were the only ones infected by IAV. The A549 cells were also the main source of IL-29 secretion. Models composed of fibroblasts and THP-1 cells in the absence of A549 cells secreted very small amounts of IL-29. This is in line with the study of Wang et al. which showed that small amounts of IL-29 can be secreted from macrophages [57].

When we infected our bioprinted lung model with influenza A virus, we observed the active replication of the virus. We then tested the effects of treatment of infected models with the anti-influenza drug oseltamivir and found a dose-dependent viral inhibition. While the lowest dose used (0.1 µM) did not have a substantial effect on viral growth, the highest concentration of 10 µM completely prevented viral growth. Intermediate concentrations of 0.5 µM and 1 µM resulted in significantly reduced viral titers after 48 h and a reduced antiviral cellular response, as indicated by the lower level of IL-29 released. For the study of viral replication in an airway chip model, Si et al. found a similar concentration of 1 µM oseltamivir to efficiently inhibit influenza viruses [58]. Nicholas et al. reported that an even lower concentration of 0.1 µM oseltamivir was effective at inhibiting influenza replication in an ex vivo model of human bronchial tissue explants [29]. In contrast, Kumar et al. used a substantially higher concentration of 100 µM of oseltamivir to inhibit the viral neuraminidase of an NS1-GFP influenza variant in A549 cells [58]. Thus, the effective dose of oseltamivir varies depending on the model system used, the cell types and the experimental setup; however, overall, the values found in our study are in the µM range reported by others as well. Oseltamivir was used as an example in the present study, as it is the most widely used anti-influenza drug in the clinic under the trade name Tamiflu. It belongs to the class of neuraminidase inhibitors; however, an increasing number of oseltamivir-resistant variants of IAV have been reported over the years. Bioprinting permits the reproducible production of organ models, which can be used for screening of new potential virus inhibitors. Efficacy of these compounds can be determined by plaque assays or quantitative RT-PCR which is sensitive and suitable for higher throughput.

The model presented here has a multi-layer structure with a base of fibroblasts and monocytes on which epithelial cells were printed to reflect the natural arrangement of the lung. Despite this progress, the model still has many limitations which need to be addressed in future studies. These include the use of cell lines. While primary fibroblasts are comparatively easy to obtain and handle, type II alveolar epithelial cells are difficult to obtain and maintain in their differentiated state. Another important point will be the cultivation of the model at an air–liquid interface (ALI) which obviously reflects the natural function of the lung. Furthermore, polarization of the epithelial cells and the inclusion of circulating macrophages will become possible with the cultivation of the model under dynamic conditions. These and other improvements will help to further increase the physiological relevance of the lung model.

To the best of our knowledge, our study reports for the first time the inhibition of a virus in an advanced bioprinted organ model by an antiviral drug in a dose-dependent manner. As outlined above, animal models of influenza infections are of limited relevance to the human course of disease due to species-specific differences [19,20,21,22,23]. Meaningful models, however, are urgently needed for the development of new drugs to treat influenza infections, as the dominating strains in recent years are often resistant to currently available compounds [59]. There is also a high medical need to develop antivirals against other viruses such as the SARS-coronavirus-2. We believe that bioprinted human organ models consisting of multiple cell types including immune cells have the potential to support these efforts by avoiding the use of xenogeneic systems in the study of human pathogenic viruses in an animal organism.

## Figures and Tables

**Table 1:** Composition of bioinks used in the present study.

	Bioink 1	Bioink 2
Alginate (% *w/v*)	2	3
Gelatin (% *w/v*)	3	3
Collagen I (mg/mL)	0.5	0.5
CaSO_4_ (M)	0.03	0.05
Fibroblasts (cells/mL)	-	2.5 × 10^7^
THP-1 (cells/mL)	-	3.5 × 10^6^
A549 cells (cell/mL)	1.5 × 10^7^	-

## Data Availability

Not applicable.

## Supplementary Materials

The following are available online at https://www.mdpi.com/article/10.3390/v13081590/s1, Figure S1: Optimization of bioink composition and culture media supplementation for 3D printed primary human normal lung fibroblast (NHLFb).
