# Supplementary material for: Bioprinted Multi-Cell Type Lung Model for the Study of Viral Inhibitors
Source: Viruses. 2021 Aug 11;13(8):1590. doi: 10.3390/v13081590 (PMC8402746; doi:10.3390/v13081590)
Supplement: Supplementary file 1 [file viruses-13-01590-s001.zip › viruses-1319459-supplementary.pdf]

Supplement

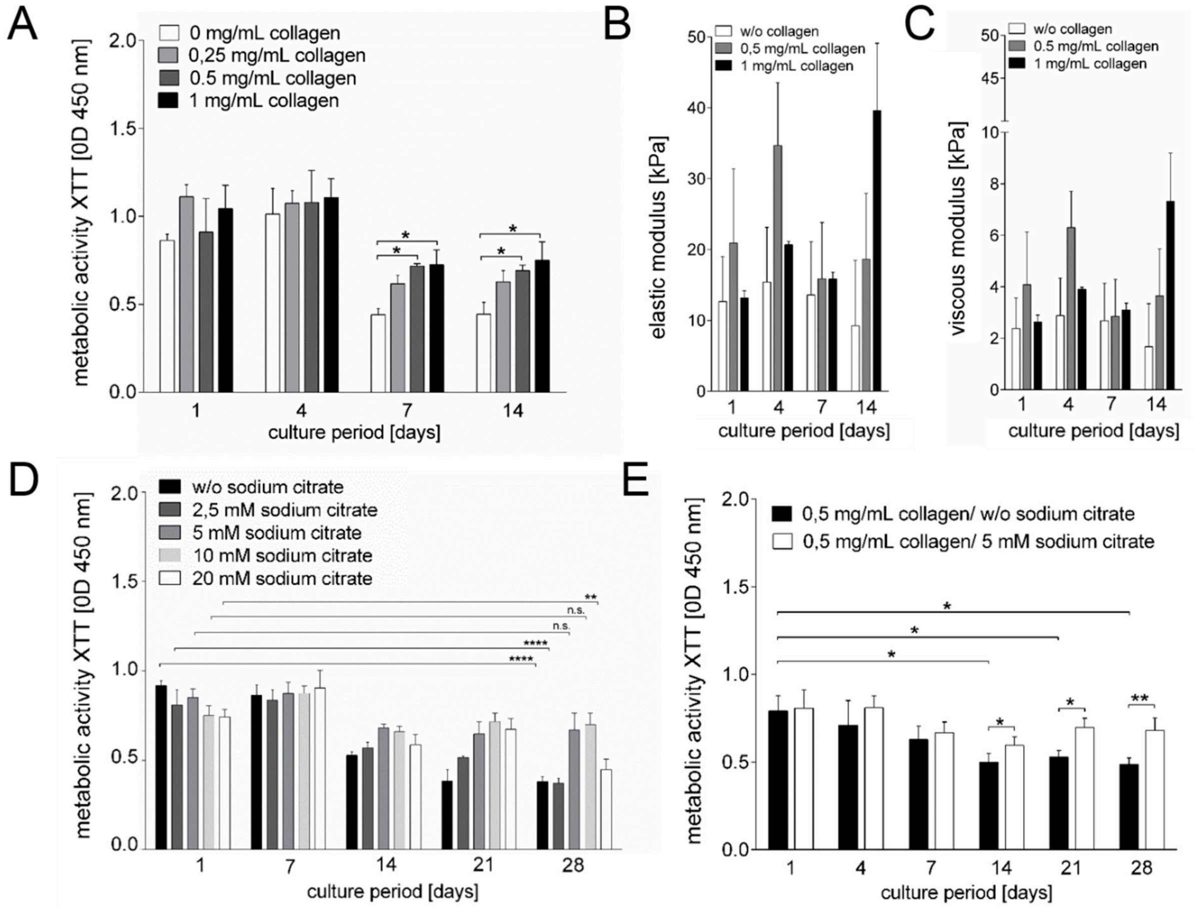

Figure S1. Optimization of bioink composition and culture media supplementation for 3D printed primary human normal lung fibroblast (NHLFb). (A) Metabolic activity of NHLFb printed in 3% alginate/ 3% gelatin bioinks with varying collagen I concentrations was determined by tetrazolium hydroxide salt (XTT) assays at the indicated time points post printing. (B and C) Rheological properties of printed alginate/gelatin constructs with varying collagen concentrations. (B) The elastic modulus of the wet bioink formulations was measured at a frequency of 1 Hz and 0.1% shear strain at 37°C at the indicated time points. (C) Shear modulus of printed constructs at increasing frequencies (0.1–10 Hz). (D; E) Metabolic activity of NHLFb printed in alginate/gelatin/0.5 mg/mL collagen bioink was determined following culture in in media supplemented with different sodium citrate concentrations by XTT assay at indicated time points post printing. Results are shown as mean ± SEM of three independent (A-D) experiment. Experiments to confirm the reproducibility of the results were carried out eight times (E). * $p \leq 0.05$; ** $p \leq 0.01$; *** $p \leq 0.001$¸**** $p \leq 0.0001$.
